# Driving Waveform Design of Electrowetting Displays Based on an Exponential Function for a Stable Grayscale and a Short Driving Time

**DOI:** 10.3390/mi11030313

**Published:** 2020-03-16

**Authors:** Zichuan Yi, Zhenyu Huang, Shufa Lai, Wenyao He, Li Wang, Feng Chi, Chongfu Zhang, Lingling Shui, Guofu Zhou

**Affiliations:** 1 College of Electron and Information, University of Electronic Science and Technology of China, Zhongshan Institute, Zhongshan 528402, China; yizichuan@163.com (Z.Y.); chifeng@semi.ac.cn (F.C.); cfzhang@uestc.edu.cn (C.Z.); Shuill@m.scnu.edu.cn (L.S.); 2 Institute of Electronic Paper Displays, South China Academy of Advanced Optoelectronics, South China Normal University, Guangzhou 510006, China; hwy956005579@outlook.com (W.H.); creekxi@163.com (L.W.); guofu.zhou@m.scnu.edu.cn (G.Z.); 3 Shenzhen Guohua Optoelectronics Tech. Co., Ltd., Shenzhen 518110, China; laishufa518@163.com

**Keywords:** electrowetting display, driving waveform, aperture ratio, exponential function, time constant

## Abstract

The traditional driving waveform of the electrowetting display (EWD) has many disadvantages, such as the large oscillation of the target grayscale aperture ratio and a long time for achieving grayscale. Therefore, a driving waveform based on the exponential function was proposed in this study. First, the maximum driving voltage value of 30 V was obtained by testing the hysteresis curve of the EWD pixel unit. Secondly, the influence of the time constant on the driving waveform was analyzed, and the optimal time constant of the exponential function was designed by testing the performance of the aperture ratio. Lastly, an EWD panel was used to test the driving effect of the exponential-function-driving waveform. The experimental results showed that a stable grayscale and a short driving time could be realized when the appropriate time constant value was designed for driving EWDs. The aperture ratio oscillation range of the gray scale could be reduced within 0.95%, and the driving time of a stable grayscale was reduced by 30% compared with the traditional driving waveform.

## 1. Introduction

In recent years, display technology has been widely used in all aspects of daily life [1,2]. In 2003, Hayes et al. proposed the EWD structure based on the principle of ink electrowetting [3], which has the advantages of low power consumption, high reflectivity, high contrast, and fast response speed. As one of new display technologies [4], increasingly more attention has been paid to EWDs. However, EWD technology has not been industrialized, and its driving technology is one of the limiting factors.

Grayscale is displayed in EWDs by applying a voltage sequence, called as driving waveform, which is used to control the form of colored ink in a pixel [5]. The ratio of a pixel area not covered by the ink to a whole pixel area is the aperture ratio, which can reflect the performance of EWDs [6]. Because the ink is divided into several parts, called the ink dispersion [7], the ink coverage area is constantly changing in the process of providing a fixed grayscale display. Therefore, an oscillation of the aperture ratio can be formed, which can reduce the number of grayscale levels and the visual experience of users. At present, some problems with EWDs are being solved, such as ink dispersion [8], charge capture [9,10], hysteresis [11], ink reflow [12], and large oscillations of the aperture ratio [13]. We have used industrial electrophoretic electronic display driver chips to drive TFT (thin-film transistor) EWDs and a four-level gray scale was realized using a PWM (pulse-width modulation) driving waveform [14]. However, the ink in the EWD pixel has obvious shrinkage and the oscillation range of the aperture ratio is relatively large due to a high switching frequency of the voltage in PWM, which has a bad effect when aiming to display stable grayscales [15]. In addition, by comparing the duty cycle of different PWMs, we found that the smaller the duty cycle of the PWM, the larger the oscillation range of the aperture ratio in the EWDs was [13]; in contrast, the larger the duty ratio, the smaller the oscillation range, which provides a reference for the driving waveform design of stable grayscales. Based on a PWM driving waveform, where the starting point of a driving waveform is set to 10 V as a maintained voltage, the optimized design of the maintained voltage and its timing can reduce the ink breakage, which can reduce the oscillation range of aperture ratio effectively; however, its driving time is extended [16]. At the same time, by optimizing the slope of the driving waveforms to control movement state of the ink in EWDs, the ink dispersion can be improved and the reflectivity can effectively be increased. However, the driving waveform increases the oscillation range of the aperture ratio and lengthens the driving time [17]. However, the damped oscillation of an electrowetting liquid drop can be optimized by changing the rising speed of the driving waveform, and the transition between the under-damping state and the over-damping state in the electrowetting lens is realized [18], which provides a reference direction for optimizing the driving waveform of the EWDs.

In order to display a stable grayscale and reduce the driving time in EWDs, an exponential-function-driving waveform was proposed in this study. By testing the impact of different time-constant values, we set parameters of the exponential function when designing the driving waveform. Compared with traditional driving waveforms, the exponential-function-driving waveform had a better driving performance.

## 2. Driving Principle of the EWD

### 2.1. Model of the EWD

**Figure 1:**
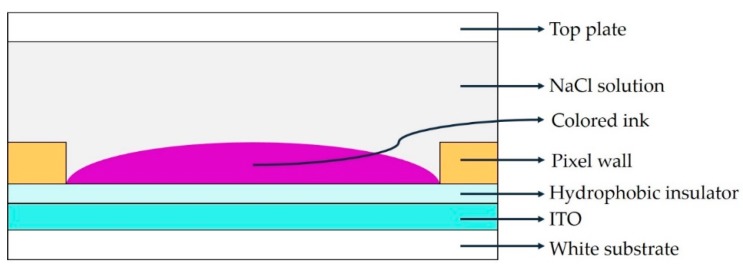
Structure of a pixel in electrowetting displays (EWDs). ITO: Indium tin oxide.

The EWD was mainly composed of a glass substrate, indium tin oxide glass (ITO), hydrophobic insulation layer, pixel wall, conductive liquid (NaCl solution), colored ink, and so on [19,20], as shown in Figure 1.

**Figure 2:**
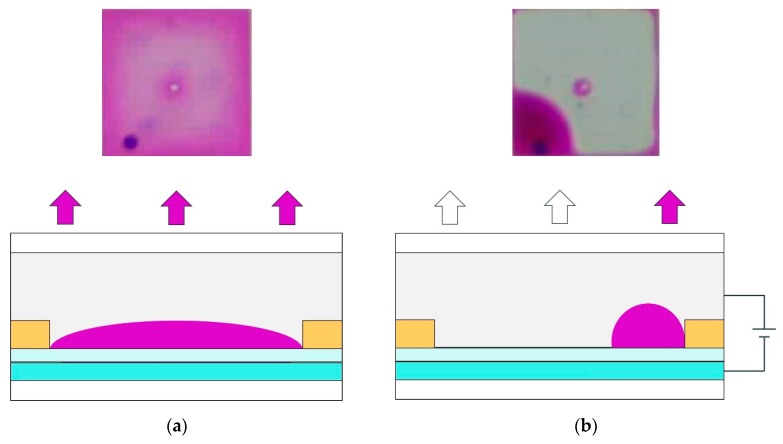
Ink state in an EWD pixel. (**a**) The pixel is “off” and the whole pixel is covered by ink such that the color of the ink is reflected. (**b**) The pixel is “on” and the ink is pushed into a corner of the pixel such that the white substrate is reflected.

The ink is laid between a NaCl solution and a hydrophobic insulation layer when no voltage is applied, and the NaCl solution does not contact the hydrophobic insulation layer directly. Then, the color of ink is displayed, and the pixel is in the “off” state, as shown in Figure 2a. The interfacial tension between the NaCl solution and the hydrophobic insulation layer is changed by an electric field force when a certain amplitude of voltage is applied between the upper and lower electrodes. At this time, the NaCl solution contacts with the hydrophobic insulating layer directly, and the ink is pushed away. Then, the pixel shows the white substrate, and the pixel is in the “on” state, as shown in Figure 2b. The ink can be spread on the hydrophobic insulating layer again when the voltage is removed such that the pixel can be continuously switched between “on” and “off “ [21].

For typical submillimeter-scale EWD pixels, the main driving forces of the ink movement are the interface tension and electrostatic force. The interface boundary is usually described using the Young–Lippmann equation [22,23], as shown in Equation (1):

(1) $$\cos\theta =\cos\theta_{0}+\frac{1}{2}\frac{\varepsilon_{0}\varepsilon_{FP}}{d\gamma_{OW}}U^{2}$$

In Equation (1), *θ* is the contact angle of the Lippmann, and *θ*_0_ is the equilibrium contact angle between the NaCl solution and the hydrophobic insulating layer. *ε_0_* represents the dielectric constant of the vacuum, *ε_FP_* represents dielectric constant of the hydrophobic insulating layer, *d* represents thickness of the hydrophobic insulating layer, *U* represents the driving voltage of the pixel, and *γ_OW_* represents the interfacial tension between the ink and the NaCl solution.

It can be seen from Equation (1) that the contact angle of the NaCl solution on the surface of the hydrophobic insulating layer can be controlled by changing the electric potential. That is to say, due to the change of electric potential energy, the surface performance of the hydrophobic insulating layer is changed, which produces a change of the conductive liquid contact angle on the hydrophobic insulating layer surface [24]. With an increase of the electric potential, the interface tension between the hydrophobic insulation layer and the NaCl solution becomes larger, the contact angle can also become larger, and the voltage difference breaks the balanced state. At this time, the NaCl solution is in direct contact with the hydrophobic insulation layer and the colored ink is pushed to a corner of the pixel to expose the white substrate. The area ratio of the exposed white substrate is called the aperture ratio, which can directly represents the reflectivity of EWDs [25], where the expression is shown in Equation (2):

(2) $$A=\left[1-\left(\frac{S_{oil}}{S_{pix}}\right)\right]\times 100\%$$

where A is the aperture ratio, *S_oil_* is the area of ink when it is pushed to a corner of a pixel, and the area of a whole pixel is *S_pix_*.

### 2.2. Design Principle of an Exponential-Function-Driving Waveform

The exponential-function-driving waveform expression is shown in Equation (3):

(3) U=U0×(1−e−tτ)

where *U* represents the real-time voltage of a driving waveform; *U_0_* represents the maximum voltage in a driving waveform, which is a fixed value; *t* represents driving time; and *τ* represents the time constant of the exponential function. We can analyze the influence of *τ* on the exponential-function-driving waveform by changing its value.

We take the derivative of Equation (3) to obtain Equation (4):

(4) U′(t)=U0τe−tτ.

When *τ* = 1 ms:

(5) $$\{\begin{array}{c} U^{\prime }\left(t\right)=U_{0}e^{-t}\\ U^{\prime }\left(1\right)=U_{0}e^{-1},\;U^{\prime }\left(2\right)=U_{0}e^{-2}\\ U^{\prime }\left(3\right)=U_{0}e^{-3},\;U^{\prime }\left(4\right)=U_{0}e^{-4} \end{array}$$

When *τ* = 6 ms:

(6) {U′(t)=U0e−t6U′(1)=U0e−16, U′(2)=U0e−13U′(3)=U0e−12, U′(4)=U0e−23

In Equations (5) and (6), *U*_0_ is a fixed value. Therefore, the change rate of the derivative in an exponential function is determined by *τ*, which is used to change the rising speed of driving waveforms.

## 3. Design of the Exponential-Function-Driving Waveform

### 3.1. Maximum Voltage of the Driving Waveform

**Figure 3:**
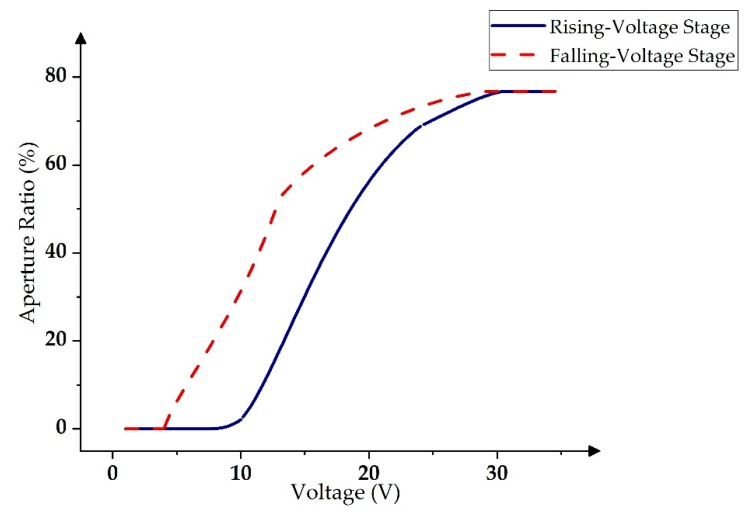
Rising-voltage stage and falling-voltage stage of the hysteresis curve in EWDs.

EWD has the characteristic of displaying hysteresis in the pixel [26,27]. In the rising-voltage stage, the ink area could be reduced slightly between 9 V and 16 V, but its aperture ratio was too small, and the pixel was not opened. However, the aperture ratio could be increased to about 50% when the voltage reached 17 V. At this time, the pixel was in an “on” state. Therefore, 17 V was the threshold voltage of a pixel. Then, the aperture ratio could reach the maximum value when the voltage was increased to 30 V. In the falling-voltage stage, the aperture ratio could be closed to 0 when the voltage was reduced to 5 V. Hence, the aperture ratio of the pixels was different between the rising-voltage stage and the falling-voltage stage at the same voltage value, as shown in Figure 3. In addition, it is easy to break through the hydrophobic insulation layer if the voltage is too high, which can cause damage to the EWD pixels [28]. Therefore, the maximum voltage of the driving waveform was set as 30 V.

### 3.2. Time Constant of the Exponential Function

**Figure 4:**
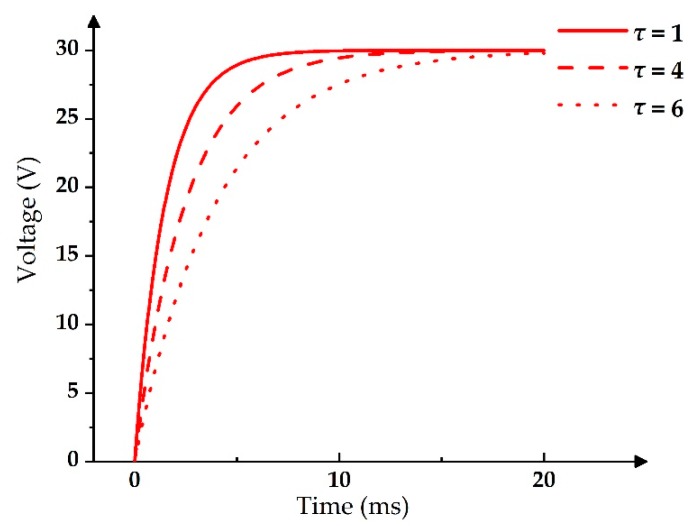
Exponential functions with different *τ* values.

By taking the derivative of the exponential function, the derivative value at each time point can be obtained. Furthermore, it can be changed by using different time constants *τ*. The smaller the value of *τ*, the greater the variation of the exponential function derivative when the frequency and the maximum voltage *U_0_* are fixed. On the contrary, the larger the value of *τ*, the smaller the exponential function derivative change is. Therefore, the voltage-rising speed can be controlled by changing the derivative, which can be used to control the oscillation range of the aperture ratio for stable grayscales and reduce the driving time. As shown in Figure 4, the exponential function with *τ* = 1 ms was the steepest one, whereas the exponential function with *τ* = 6 ms was the smoothest one. The exponential function was close to PWM due to its fast voltage-rising speed when *τ* < 1 ms, and it could not reach the maximum driving voltage in a driving cycle due to its slow voltage-rising speed if *τ* > 6 ms at the same time. Therefore, the value of *τ* was in the range of 1 ms ≤ *τ* ≤ 6 ms.

**Figure 5:**
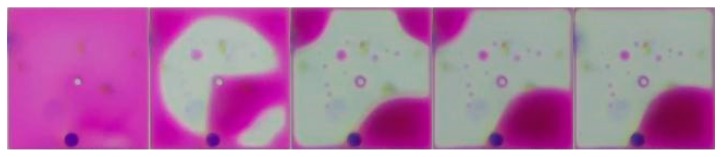
The ink formation change process in the driving process of a pixel.

Ink in the pixel can be pushed toward corners using a conductive NaCl solution by applying a driving voltage to EWDs when a grayscale needs to be displayed. However, the ink may be divided into several parts in this process; this phenomenon is called ink dispersion [29]. With the increase of the driving time, some smaller inks can gradually move closer to the larger one, as shown in Figure 5. In this process, the grayscale is unstable until all the ink is in one corner of a pixel.

**Figure 6:**
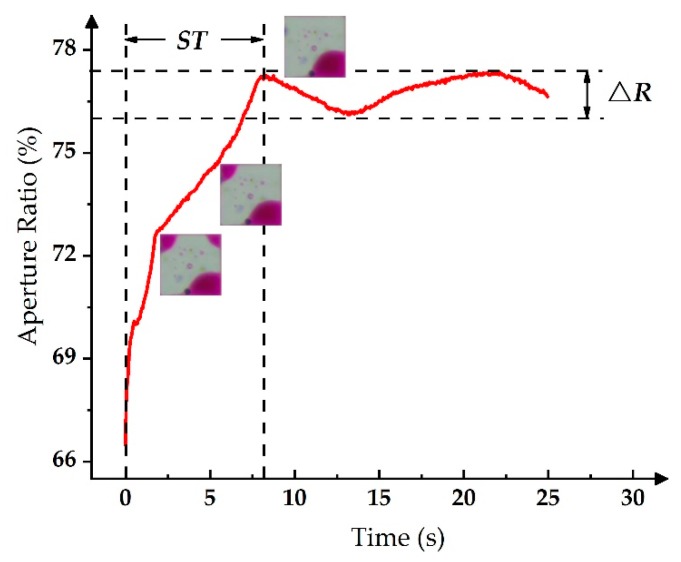
The aperture ratio change process when a driving waveform is applied to the EWD.

Therefore, there is an oscillation phenomenon of the aperture ratio in all driving processes, which has a bad effect when aiming to display stable grayscales. The aperture ratio of the pixel decreased from 77% to 76%, and then increased to 77%, where the oscillation range of the aperture ratio is expressed as ∆*R* in this paper. In addition, the driving time for a stable grayscale is expressed as *ST*, where a complete driving process is shown in Figure 6.

**Figure 7:**
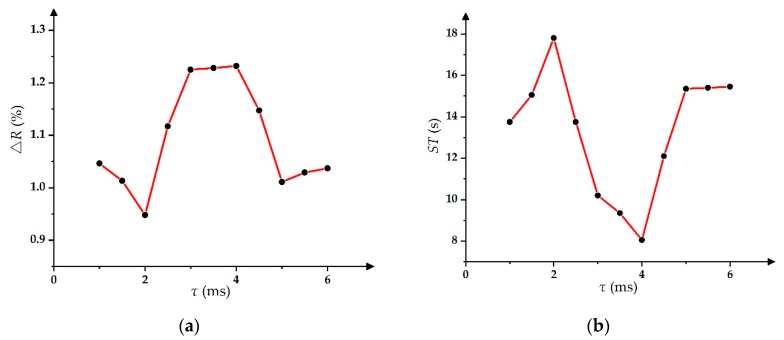
The relationship among parameters of the exponential-function-driving waveform. (**a**) The relationship between *τ* and ∆*R*. (**b**) The relationship between *τ* and the stable time.

Then, the relationship between *τ* and ∆*R* can be tested by using the exponential-function-driving waveform to drive the EWD, and the same method can be used to test the relationship between *τ* and *ST*. In the testing, the value range of *τ* was from 1 ms to 6 ms, where the relationship curves are shown in Figure 7.

As shown in Figure 7a, with the increase of *τ*, ∆*R* reached the minimum value of 0.95% when *τ* = 2 ms. Furthermore, ∆*R* reached the maximum value of 1.25% when *τ* = 4 ms. As shown in Figure 7b, with the increase of *τ*, the ST reached the minimum value of 8 s when *τ* = 4 ms, where the maximum *ST* was 18 s when *τ* = 2 ms.

## 4. Experimental Results and Discussion

### 4.1. Testing System

In order to test the effect of driving waveforms, we developed an experimental platform to record the aperture ratio and the driving process to measure the oscillation range of the aperture ratio and the driving time of the grayscales. The driving system consisted of a computer, a waveform generator and a high-voltage amplifier, which were used to edit the driving waveform for the EWD. The testing system consisted of a microscope and a high-speed camera. Relevant equipment information is shown in Table 1. Furthermore, an EWD panel was used in the experiment, where its parameters are shown in Table 2.

In the EWD panel, the hydrophobic insulation layer was not resistant to a high voltage, and the withstand voltage value was under 40 V. The hydrophobic layer material was Teflon AF1600, and its solution was FC-43, which is a fluorocarbon solvent from the 3M company (Maplewood, MN, USA); the pixel wall grid material was transparent polyimide; the ITO glass substrate came from Shenzhen Laibao Hi Tech Co., Ltd. (Shenzhen, China); the glass thickness was 0.7 mm and the impedance is 100 Ω/cm^2^; and the electrolyte solution was NaCl with a concentration of 1 × 10^−4^ mol/L. Deionized water was obtained using an ultra-pure ultraviolet water purification system.

In the testing process, the driving waveform was edited using MATLAB (Mathworks, Natick, MA, USA) and ArbExpress software (V3.4, Tektronix, Ohioan, USA), and then it was sent to the signal generator via a serial port. Since the maximum output voltage of the signal generator was 5 V, the EWD could not be driven by the signal generator directly. Therefore, the signal generator should be connected with a high-voltage amplifier for outputting the driving voltage. In addition, an industrial camera was used to record the ink state of the EWD in real time using a microscope. The microscope could magnify the pixel 200 times and the resolution of the industrial camera was 1920 × 1080. The real-time picture of a pixel could be captured every 50 ms and the aperture ratio of the pixels could be calculated for each picture using binary processing. Therefore, the relationship between the aperture ratio and the time could be obtained. During the experiment, the surrounding temperature was 25 °C and the humidity was 60%. Next, two comparative experiments are shown as follows.

### 4.2. Oscillation Range of the Aperture Ratio

**Figure 8:**
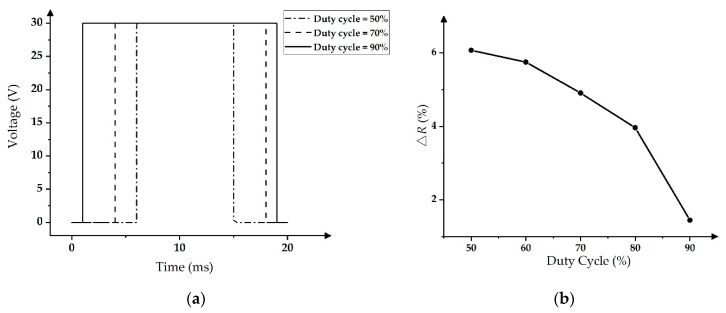
The pulse width modulation (PWM) driving waveform and its aperture ratio performance [14,30]. (**a**) The PWM driving waveform with different duty cycles. (**b**) Relationship between the duty cycle and ∆*R*.

Two traditional driving waveforms were used to analyze and compare the oscillation range ∆*R*. In the design of the traditional PWM driving waveform, the maximum voltage was still set to 30 V, and its duty cycle was changed from 50% to 90%, as shown in Figure 8a. The smaller the duty cycle, the smaller the ratio of the maximum voltage in an entire driving cycle.

The testing results of ∆*R* in PWM are shown in Figure 8b; as the duty cycle increased, ∆*R* decreased. The ∆*R* was as high as 6.1% when the duty cycle was at 50%, and the ∆*R* was reduced to 1.4% when the duty cycle was at 90%, which met the requirement for stable grayscales. However, the ink in a pixel reflowed when the duty cycle was larger than 90% and the pixel could be closed at this time [14,30]. Therefore, this situation was meaningless for the experiment.

**Figure 9:**
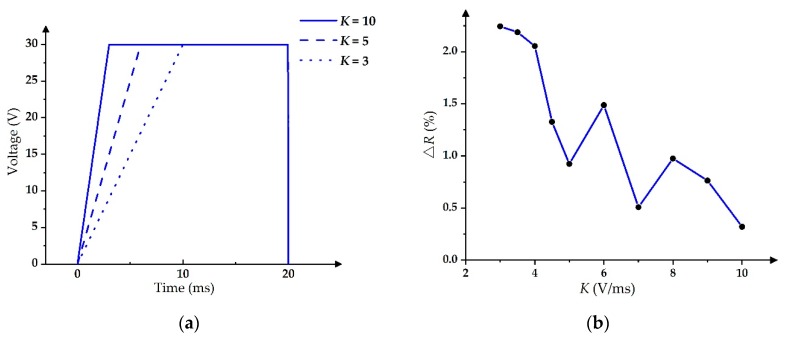
Slope-driving waveform and its aperture ratio performance [17,31]. (**a**) Slope-driving waveform with different *K* values. (**b**) Relationship between *K* and ∆*R*.

In the contrast experiment of slope-driving waveforms, the maximum driving voltage was set to 30 V and the slope value range of driving waveforms was from 3 V/ms to 10 V/ms, as shown in Figure 9a, where *K* is the slope [17,31]. Furthermore, the relationship between *K* and ∆*R* is shown in Figure 9b, where the maximum value of ∆*R* was 2.2% when *K* was 3 V/ms; the minimum value of ∆*R* was 0.3% when *K* was 10 V/ms.

**Figure 10:**
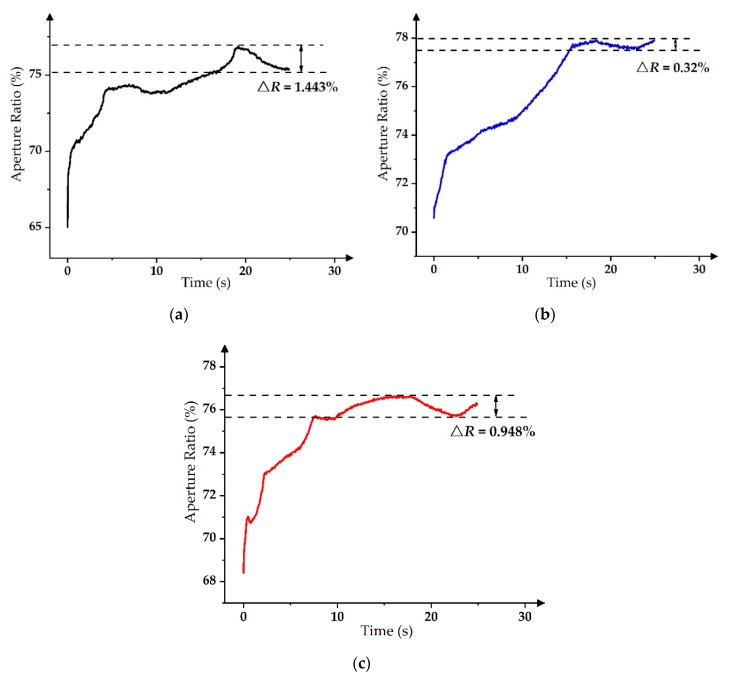
Aperture ratio response of different driving waveforms. (**a**) PWM with a duty cycle of 90% [14,30]. (**b**) Slope-driving waveform with *K* = 10 V/ms [17,31]. (**c**) Exponential function with *τ* = 2 ms.

In order to compare ∆*R* among three kinds of driving waveforms, the parameters of three driving waveforms were optimized to obtain the minimum value of ∆*R*. The minimum ∆*R* of the PWM was 1.4% when the duty cycle was 90%, the minimum ∆*R* of the slope-driving waveforms was 0.3% when *K* was 10 V/ms, and the minimum ∆*R* of the exponential-function-driving waveform was 0.95 when *τ* was 2 ms. As shown in Figure 10, the PWM could cause a large grayscale oscillation but the slope-driving waveform and the exponential-function-driving waveform could solve the problem. As a result, more levels of the grayscale display could be achieved.

### 4.3. Driving Time of a Stable Grayscale

**Figure 11:**
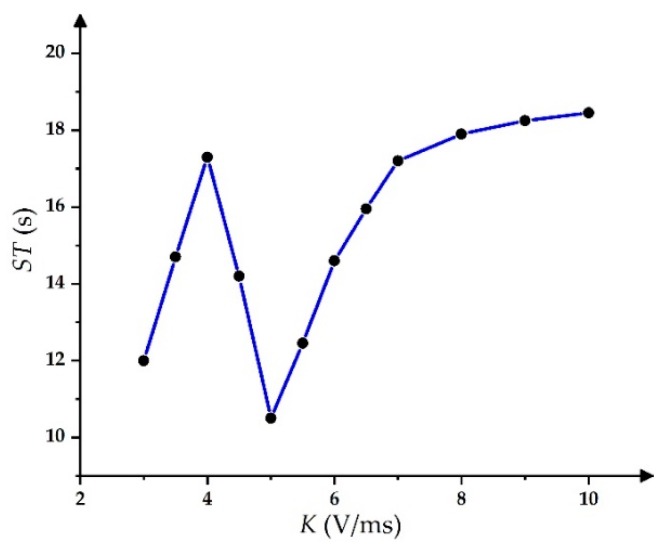
The relationship between *ST* and the slope of the driving waveform.

In the PWM driving waveform, ∆*R* was 1.4% when the duty cycle was 90%, and its *ST* for stable grayscales was 19 s. Furthermore, ∆*R* values of other duty cycle PWM waveforms were greater than 3%; therefore, the PWM driving waveform with a 90% duty cycle was the best one. In the slope-driving waveform, the *ST* could reach the minimum value when *K* was 5 V/ms, as shown in Figure 11.

**Figure 12:**
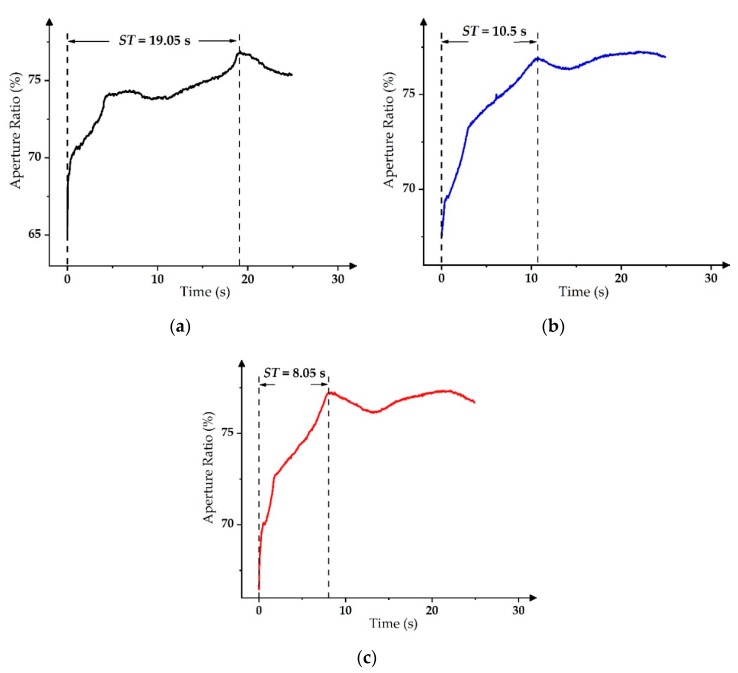
Driving time for the stable grayscale display of different driving waveforms. (**a**) The PWM with a duty cycle of 90% [14,30]. (**b**) The slope-driving waveform with *K* = 5 V/ms [17,31]. (**c**) The exponential function with *τ* = 4 ms.

In the exponential-function-driving waveform, *ST* was the shortest when *τ* was 4 ms. As shown in Figure 12, the *ST* of the PWM driving waveform was 19 s. Furthermore, the *ST* of the slope-driving waveform could be shortened to 10.5 s. However, the *ST* of the exponential-function-driving waveform was the shortest with 8 s when *τ* was 4 ms. Therefore, the driving time for stable grayscales could be reduced by using the exponential-function-driving waveform, and the oscillation of the aperture ratio could be restrained in a certain range at the same time. Then, it could be used to effectively improve the static display ability of EWDs.

## 5. Conclusions

In order to reduce the oscillation of the aperture ratio and the driving time for stable grayscales in pixels, an exponential-function-driving waveform was proposed for improving the performance of EWDs. Then, the time constant of the exponential function was optimized by testing the driving process of the aperture ratio. The results showed that the grayscale oscillation could be controlled in a certain range by using the exponential-function-driving waveform when its time constant was 2 ms, which could be used to reduce the flicker of EWDs. In addition, the shortest driving time of the stable grayscale could be obtained by using the proposed driving waveform when its time constant was 4ms, which could improve the static display performance of EWDs.

## Figures and Tables

**Table 1:** Experimental platform for testing EWDs.

Category	Computer	Waveform Generator	High Voltage Amplifier	Microscope	Industrial Camera
Manufacturer	Lenovo	Tektronix	Agitek	Cossim	Koppace
Model	M425	AFG-3052C	ATA-2022H	SZ760T2LED	KP-AF200

**Table 2:** Parameters of the EWD panel.

Panel Size (cm)	Ink Color	Ink Thickness (µm)	Resolution	Pixel Size (μm^2^)	Pixel Wall Height (µm)	Pixel Wall Size (μm^2^)	Hydrophobic Layer Thickness (µm)
3.5 × 3.5	Purple	5	50 × 50	400 × 400	6	15 × 15	1
